# Prevalence of Epileptiform Discharges in Children with Sensori-Neural Hearing Loss and Behavioral Problems Compared to Their Normal Hearing Peers

**Published:** 2014

**Authors:** Susan AMIRSALARI, Shokoufeh RADFAR, Mohammad AJALLOUYEAN, Amin SABURI, Jaleh YOUSEFI, Sima NOOHI, Seyed Abbas TAVALLAIE, Mahdieh HASSANALIFARD, Yasaman GHAZAVI

**Affiliations:** 1 New Hearing Technologies Research Center, Baqiyatallah University of Medical Sciences, Tehran, Iran; 2 Chemical injuries Research Center, Baqiyatallah University of Medical Sciences, Tehran, Iran; 3 Behavioral Science Research Center, Baqiyatallah University of Medical Sciences, Tehran, Iran; 4 Shahid Beheshti University of Medical Sciences, Tehran, Tran

**Keywords:** Sensorineural hearing loss, Overactivity and behavioral problems, Electroencephalography, Epileptiform discharges

## Abstract

**Objective:**

Overactivity and behavioral problems are common problems in children with prelingually profound sensorineural hearing loss (SNHL). Data on epileptiform electroencephalography (EEG) discharges in deaf children with psychological disorders are so limited. The primary focus of this study was to determine the prevalence of epileptiform discharges (EDs) in children with SNHL and overactivity or behavioral problems.

**Materials & Methods:**

A total of 262 patients with prelingually profound SNHL who were referred to our cochlear implantation center between 2008 and 2010 were enrolled in this study. Children with SNHL who had diagnosis of overactivity and/or behavioral problems by a pediatric psychiatrist, underwent electroencephalography (EEG).

EEG analysis was carried out by a board-certified pediatric neurologist. The control group consisted of 45 cases with overactivity or behavioral problems and normal hearing.

**Results:**

One hundred thirty-eight children with mean age of 3.5±1.23 year were enrolled in the case group, of whom 88 cases (63.7%) were boy. The control group consisted of 45 cases with mean age of 3.2±1.53 years, of whom 30 (66.6%) cases were male. EDs were detected in 28 (20.02%) children of the case group (with SNHL) in comparison with 4 (8.88%) in the control group (without SNHL), which was statistically significantly different.

**Conclusion:**

In this study, we obtained higher frequency of EDs in deaf children with overactivity and/or behavioral problem compared to the children without SNHL.

Further studies are required to evaluate the possible association of SNHL with EDs in overactive children.

## Introduction

Overactivity and behavioral problems is one of the most common psychiatric disorders of childhood. The overall incidence of overactivity and behavioral problems is about 3-8% of children with some deviations in different countries (1). Lack of a single etiology, lack of differentiation from other behavioral and learning disorders, and lack of consistent response to treatment have made it difficult to establish a distinct syndrome. The incidence of attention deficit/hyperactive disorder in children with inherited deafness seems to be like the normal range in the society, while it seems to be higher in children with acquired deafness and/or additional problems (2). A re-analysis of an earlier study revealed that psychiatric disorders are more common in deaf children than expected (2). The diagnosis of overactivity and ADHD is made according to the DSM IV criteria, of which the essential characteristic includes a consistent pattern of lack of attention and/or hyperactivity-impulsivity more frequent and severe compared to that typically seen in subjects with an equivalent developmental level (3).

There are new trends toward epileptiform activity in electroencephalography (EEG) as a diagnostic clue for ADHD and overactivity disorder. Various research studies have demonstrated this association, suggesting that epileptiform EEG abnormalities might be a good clue in diagnosis of overactivity and ADHD (1,4- 7). Some studies have affirmed that EEG data allow differentiating between overactive and normal children with a specificity of 94% and sensibility of 90% (8,9). 

Other studies indicate the value of EEG in hyperkinetic disorders diagnosis (10,11), but there is still not sufficient evidence to use EEG as a routine diagnostic method for childhood overactivity or other behavioral disorders (12). On the other hand, there are a few data which suggest the possibility of relationship between primary developmental language disorders and abnormal EEG results (13). Although cochlear implantation was used successfully for epileptic children with sensori-neural hearing loss (SNHL), it is possible that the outcome would be affected in children with additional disabilities such as psychiatric disorders (14,15). The existing evidences are not sufficient to use EEG as a routine screening method for children with SNHL who are candidate for cochlear implant. The primary focus of this study was to determine prevalence of epileptiform EEG discharges in patients with overactivity and/or behavioral problems with concurrent SNHL. 

## Materials & Methods

A total of 262 cochlear implant candidates with prelingually profound hearing los (aged 1-4 years) were enrolled in this historical case-control study. All of the cases were referred to our cochlear implantation center from June 2008 to February 2010. After primary evaluation, all cases were seen by a pediatric psychiatrist, and 138 patients with diagnosis of overactivity and/ or behavioral problems underwent EEG evaluation, and prevalence of epileptiform discharges (EDs) were measured by the same pediatric epileptologist.

The inclusion criteria for these patients were as follows: 

1) permanent SNHL, 2) onset of hearing loss before 6 months of age, 3) use of amplification and/or intervention program emphasizing spoken language, 4) Persian as the language of intervention. The exclusion criteria were: 1) history of convulsive disorders, 2) history of CNS diseases, and 3) history of mental retardation or autistic spectrum disorder. Considering the fact that all cases were under 4 years of age and had overactivity or behavioral problems, the EEG was recorded in sleep while the subjects were sedated by chloral hydrate to avoid movement artifacts. The electrodes were applied based on the international 10-20 System. The sleep rhythm and also the type, location, and side of epileptiform activities were assessed by an epileptologist. Finally, the prevalence of epileptiform EEG discharges in these deaf children was compared with a group of normal hearing children with overactivity and/or behavioral problems consisting of 45 cases with matched age and sex distribution and enrollment criteria. Our study was approved by the Ethics Committee of Baqiyatallah University of Medical Sciences, and informed written consents were taken from all the patient’s parents.

## Results

One hundred thirty-eight candidates of cochlear implantation with prelingually profound hearing loss and mean age of 3.5±1.23 years were evaluated in this study. Eighty-eight cases were boys (63.7%) and the remaining 50 cases were girls (36.3%). The control group consisted of 45 cases with overactivity and/or behavioral problems and normal hearing with mean age of 3.2±1.53 years and sex distribution of 30 boys (66.6%) and 15 girls (34.4%). There were not significant differences between these two groups with respect to sex and age (p=0.51). Epileptiform activity was diagnosed by the appearance of generalized or focal paroxysmal spike and wave or sharp and wave complexes in the tracing. 

**Fig 1:**
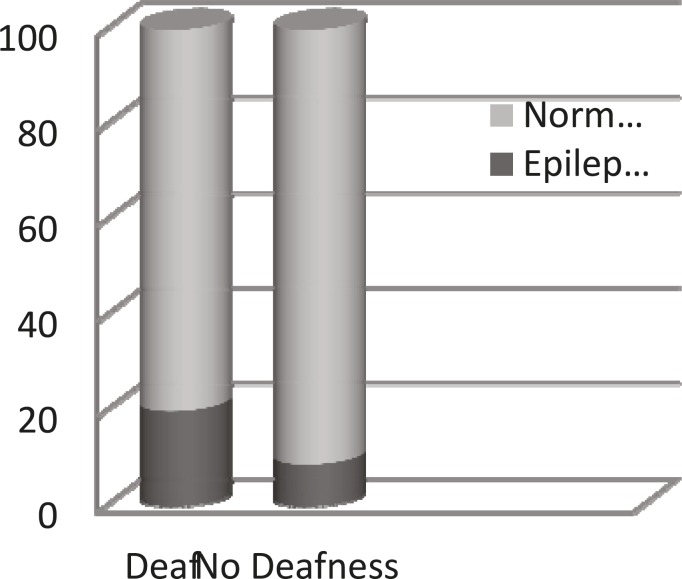
Frequency of epileptiform EEG discharges in children with behavioral problems and/or overactivity in normal hearing versus deaf peers

EDs were recorded in 28 (20.02%) patients in the case group compared to 4 (8.88%) patients in the control group. These children did not suffer from epileptic fits. There was no association between epileptiform activity and age, gender, or use of hearing aids in both groups. There was a significant increase in epileptiform EEG discharges in our case group (overactivity and/ or behavioral problems+sensorineural hearing loss) in comparison with the control group (overactivity and/or behavioral problems+normal hearing ability) (p= 0.01) (Figure 1).

## Discussion

In the present study, the finding of EDs in 28 (20.02%) children with overactivity and/or behavioral problems with concomitant deafness is much higher than the values of 6.1%, 5.6%, and 7.5% in children with overactivity and/or behavioral problems and normal hearing ability found in other literature (16-18) and higher than that found in healthy children (2%-6.5%) (19,20). 

Furthermore, this value of EDs in the case group (20.02%) was much higher than that in the control group (8.88%). This higher prevalence highlights the role of EEG evaluation in patients with overactivity and/or behavioral problems and hearing loss. The main theme arising from the results of this study is that EEG evaluation could be a good predictor for diagnosis of overactivity and/or behavioral problems in children with concomitant hearing loss compared to normal hearing children.

Fewer alpha waves and more delta and fast theta waves were observed in Matsuura et al.’s study on children with ADHD in psychiatric clinics in Japan, China, and Korea (1). Eighty-three percent sensitivity for epileptiform EEG abnormality in diagnosis of hyperkinetic disorders was found in Fonesca et al.’s study on 30 ADHD and 30 control cases with no neurological or psychiatric problems (5). Magee et al., in a study using similar procedures affirmed 89.0% sensibility and 79.6% specificity (11). Monastra et al. affirmed that EEG data allow for differentiation between hyperkinetic children and normal children with a specificity of 94% and sensitivity of 90% (8,9). Although, many studies have demonstrated EEG epileptiform abnormalities might be a good clue for diagnosis of hyperkinetic and behavioral disorders, the clinical use of routine EEG in children with overactivity and/or behavioral problems appears to be limited and its recommendation depends on the suspicion of epileptic manifestations and it is not still a routine diagnostic method (20).

There is no established evidence of higher EDs in deaf children in literature, which decreases the possibility of isolated hearing loss as a principle cause of higher prevalence of ED in deaf children with overactivity and/ or behavioral problems. Existence of epileptiform waves in EEG and overactivity or behavioral problems as an additional disorder may affect the auditory rehabilitation such as cochlear implantation, although there is no previous report with focus on this issue (21,22).

The present study had some limitations, which restricted us to make final conclusion: first, the small sample of controls, which had epileptifirm waves, limited us to statistically compare them with SNHL cases; and second, if the outcome of treatment such as cochlear implantation would be compared between the two groups, it could be useful for further study, although we did not follow the cases.

No research studies have been performed to evaluate epileptiform EEG discharges in deaf children with concurrent overactivity and/or behavioral problems, but in this study, we obtained higher frequency of EDs in deaf children with overactivity and/or behavioral problem in comparison with the children without SNHL. 

**In conclusion,** we obtained higher frequency of EDs in deaf children with overactivity and/or behavioral problem compared to the children without SNHL. 

Further studies are better to perform on evaluating the possible association of SNHL and EDs in overactive children.
